# Just the Facts: Protecting frontline clinicians during the COVID-19 pandemic

**DOI:** 10.1017/cem.2020.359

**Published:** 2020-04-02

**Authors:** Paul Atkinson, James French, Eddy Lang, Tamara McColl, Laurie Mazurik

**Affiliations:** * Department of Emergency Medicine, Dalhousie University, Saint John Regional Hospital, Saint John, New Brunswick; † Office of the Chief Medical Officer, WorkSafeNB, Saint John, NB; ‡ Department of Emergency Medicine, University of Calgary, Calgary, AB; § Department of Emergency Medicine, University of Manitoba, Winnipeg, MB; ¶ Department of Emergency Medicine, University of Toronto, Sunnybrook Health Sciences Centre, Toronto, ON

**Keywords:** Coronavirus, emergency department, hazard control measures, personal protective equipment

## Abstract

There is no patient emergency more important than protecting health care workers during a pandemic.

## CLINICAL SCENARIO

There is no patient emergency more important than protecting health care workers during a pandemic.

You are an emergency physician looking for clear advice on protecting frontline emergency department (ED) clinical staff during the coronavirus disease (COVID-19) pandemic. You turn to trusted sources for advice and set about summarizing the best available evidence into a useful format to help you and your staff minimize and mitigate the risks of contracting coronavirus infection while treating patients in the ED during the pandemic.

## KEY CLINICAL QUESTIONS

1.: **Are emergency services staff at risk of contracting coronavirus and becoming ill during the COVID-19 pandemic?**

Yes. The presence of novel coronavirus or SARS-CoV-2 (severe acute respiratory syndrome coronavirus-2) in the community poses a real and evident risk to all health care workers, especially those caring for symptomatic and infected patients. Whether contracting the disease from asymptomatic unidentified patients, or from symptomatic suspected cases, it is clear that health care workers have a significant risk of morbidity from the virus, and unfortunately a growing number of health care worker deaths are being attributed to the disease internationally.^1^ During the SARS epidemic in 2003, health care workers made up a significant number of infected cases.^2^ However, it is encouraging that compliance with testing and infection prevention and control procedures in Hong Kong during the early phases of the COVID-19 pandemic seemed to be effective in preventing infection in health care workers.^3^

2.: **What measures other than personal protective equipment (PPE) should be implemented to reduce the risk of frontline staff contracting the virus?**

**Figure 1.**
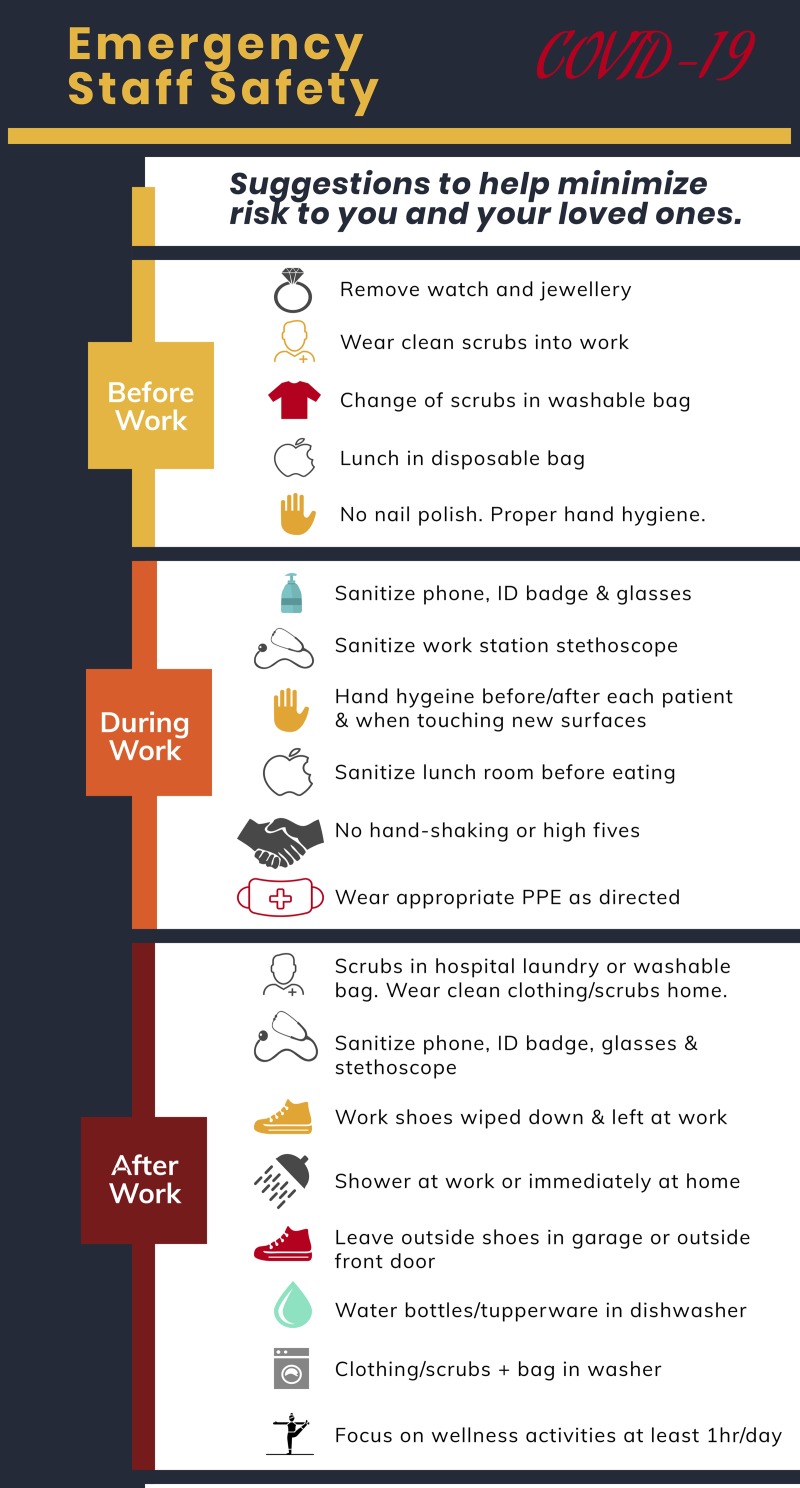
Infographic detailing general hygiene measures for emergency department staff.

Minimization of exposure to the virus is key. According to the World Health Organization (WHO)^4^ and the Public Health Agency of Canada (PHAC)^5^ *general measures* that apply to the population, both patients and health care workers, should be universally implemented (Figure 1). Simple routines can help decrease provider risk, including removing all jewelry and watches during patient care; not wearing nail polish; performing frequent hand hygiene with an alcohol-based hand wash or with soap and water for at least 20 seconds; avoiding touching your face; sanitizing your work station, stethoscope, and phone throughout your shift; practicing respiratory hygiene; and maintaining physical distance (a minimum of 2 meters) from persons with respiratory symptoms, or anyone who is at risk of being infected. Additionally, changing into clean clothing before leaving work and showering in hospital or immediately upon arrival to your home will also help protect you and your family. Failure to follow these general measures is associated with higher rates of infection.^6^ Health care workers who start exhibiting infectious symptoms should immediately self-isolate and contact their administrators to discuss COVID-19 testing.

In addition, targeted *administrative, environmental, and engineering controls* should be implemented to reduce the risk of exposure. The National Institute for Occupational Safety and Health (NIOSH)^7^ outline a hierarchy of controls. Health care organizations should ensure input from infection prevention and control (IPC) specialists to ensure appropriate infrastructure, testing capacity, zoning of patients according to risk of disease, and adequate staffing levels and training. Engineering interventions, such as physical separation of staff from patients where possible (e.g., the use of telephone or intercom communications) and appropriate ventilation (e.g., negative pressure rooms for aerosolizing procedures), are important in minimizing the viral exposure to health care workers. Realistically, many EDs will not be equipped with the “recommended” infrastructure or equipment, but reasonable alternatives exist. These may include identifying walled rooms for aerosol-generating medical procedures (AGMPs) with appropriately sized high-efficiency particulate air (HEPA) filters, taping off donning/doffing areas at least 2 meters from the patient, communicating with patients/teams using walkie-talkies or baby monitors, requiring patients to wear face masks, and establishing “clean/cold” and “dirty/hot” areas of the department with respect to suspected or confirmed COVID-19 patients with dedicated staff in each area if resources allow.

3.: **What PPE is recommended when treating potential COVID-19 patients?**

Recommendations for the appropriate levels of PPE required by health care workers tend to vary by administrative regions, by risk of exposure and procedure, and with time as evidence is reviewed and incorporated into guidelines. Droplet and contact precautions are now recommended for the routine care of all ED patients with suspected or confirmed COVID-19; however, due to the poor reliability of clinical features in predicting cases, some jurisdictions, including Canada, have now implemented universal precautions for *all* ED patients.^5^ Airborne precautions should be used when AGMPs are planned or anticipated to be performed on all patients during the pandemic.^8^ WHO and PHAC provide guidance on the minimum levels of protection required for various situations, including triage of undifferentiated patients, standard treatment of suspected cases and also for high risk procedures where aerosolization of the virus is most probable.^4^ Their advice covers respirators (e.g., N95, FFP2, or equivalent standard), surgical masks, eye and face protection, water resistant and waterproof gowns, and gloves (Figure 1). Other regional occupational health and safety (OSH) agencies provide locally applicable standards.^8^ It is clear that, when in close contact with a patient suspected or diagnosed with COVID-19, in addition to minimizing the time with the patient, health care workers should be provided with appropriate levels of protection, such as respirators and eye and face protection, if the severity of the case is undetermined (e.g., at triage), or there is any risk of airborne (aerosol) spread, such as during resuscitation, noninvasive ventilation, high flow nasal cannulae, airway procedures, or if the patient is actively coughing into the air.

Similarly, gowns should be waterproof (Level 2, fluid resistant, or higher) must have full length sleeves, and should cover as much of the workers body as possible, including neck and back. Full face and eye shields should be worn to avoid mask/respirator and face contamination. The combination of a face shield and goggles or industrial glasses may fog, so choose wisely. The use of face shields is particularly important in protecting supplies of respirators by preventing contamination. If you have a hood, wear it. It is sensible to cover as much of the head and hair as possible during high-risk aerosolizing procedures or situations. Innovative techniques to preserve and augment standard PPE are emerging. As community spread becomes established, all patients, regardless of their presentation, should be seen as potentially infectious, and health care workers should ensure that they wear universal precautions, and practice hand hygiene and appropriate distancing at all times in the ED.

4.: **How important is training and monitoring in preventing infection?**

Very important. A guideline is only as effective as the way in which it is implemented and adhered to. While concerns about supplies of PPE and the reliability of PPE recommendations are valid, these conversations can distract from the importance of compliance with good practice when it comes to wearing and removing (donning and doffing) PPE. While careful donning of PPE is important, the higher risk of worker contamination is when removing the PPE during doffing. Appropriate training, by video^9^ and in person with a buddy system, drills, checklists, and in situ simulation can greatly enhance the effectiveness of PPE use. Managers tasked with oversight of worker health and safety should personally participate in training and practice sessions for selecting, donning, and doffing of PPE, in addition to regular observation and coaching on shift. Situations where breaches in PPE procedures are most likely include those where there is insufficient warning or time to prepare for a critical procedure, such as cardiac or respiratory arrest or rapid decline of a patient's clinical status. It is vital that emergency health care workers, either in hospital or in the community take the time to don appropriate PPE before initiating treatment, no matter how urgent the situation may be.^10^ The health and safety of health care workers is primary in a pandemic, to be able to continue to provide care for subsequent patients.

5.: **Is there a role for staff screening and testing during the pandemic?**

Individual jurisdictions will have different policies and procedures for staff screening and testing. What is clear is that an active screening procedure for staff, preventing symptomatic or potentially infected staff from working among well staff and patients is vital in preventing super-spreaders. Screening questions about travel, contacts, contamination events, and symptoms are the minimum required, with temperature checks for staff before each shift providing additional screening power. Self-isolation, self-monitoring, and testing of all exposed or symptomatic staff members is important in preventing disease spread. Expedited results in consultation with the local infectious disease department are paramount to maintain frontline staffing. It can be difficult to define exposure in frontline staff; however, close contact with a high-risk or positive case without wearing the appropriate standard of PPE is a reasonable definition.

6.: **Are there any additional measures that should be considered for vulnerable staff members?**

Certain groups of society are at increased risk from coronavirus. This is also true among health care workers, and includes older adults (e.g., those over 60 years), those with underlying medical conditions (e.g., heart disease, chronic respiratory diseases, cancer), and those at risk due to a immunocompromise from a medical condition or treatment (e.g., chemotherapy).^5^ Mortality rates have been cited as 10 times higher in patients between the ages of 60 and 69 and over 25% in age groups over 70 years. Pregnant staff are also a consideration, as literature is sparse but suggests they can have a severe clinical course with adverse fetal effects.^11^ It seems wise then to protect these groups of health care workers from direct exposure to high-risk procedures, and perhaps restrict these staff to taking care of the lower risk stream of non–COVID-19 related cases in the ED.

## CONCLUSION

To ensure the sustained delivery of emergency care during the COVID-19 pandemic, it is essential to protect frontline health care workers from contracting the disease. Various measures, such as hazard controls, PPE, training and compliance, and protection of vulnerable individuals should be included in recommendations. In addition, careful scheduling of staff, as well as consideration for psychological and peer support and wellness initiatives are strongly encouraged.

**Additional Resources**

The Canadian Association for Emergency Physicians (CAEP; https://caep.ca/covid-19/) and the International Federation for Emergency Medicine (IFEM; https://www.ifem.cc/coronavirus-2019-information/) provide additional updated resources on this topic.

**KEY POINTS**

1. General measures such as handwashing and distancing are vital.
2. Spend as little time as possible in close proximity to suspected or confirmed COVID-19 patients.
3. Use administrative and engineering controls to protect staff from exposure when possible.
4. Wear appropriate PPE – respirators, face shields, waterproof gowns, and gloves for all situations where aerosolization of the virus is possible. Consider head protection.
5. Do not waste PPE supplies; each piece of equipment should be used for as long as is safe and effective.
6. Consider extra protections for high-risk groups.
7. Think about mental health and wellness—be kind and supportive to your team.
